# FlbB forms a distinctive ring essential for periplasmic flagellar assembly and motility in *Borrelia burgdorferi*

**DOI:** 10.1371/journal.ppat.1012812

**Published:** 2025-01-08

**Authors:** Jack M. Botting, Md Khalesur Rahman, Hui Xu, Jian Yue, Wangbiao Guo, Joshua T. Del Mundo, Michal Hammel, Md A. Motaleb, Jun Liu

**Affiliations:** 1 Microbial Sciences Institute, Yale University, West Haven, Connecticut, United States of America; 2 Department of Microbial Pathogenesis, Yale School of Medicine, New Haven, Connecticut, United States of America; 3 Department of Microbiology and Immunology, Brody School of Medicine, East Carolina University, Greenville, North Carolina, United States of America; 4 Molecular Biophysics and Integrated Bioimaging Division, Lawrence Berkeley National Laboratory, Berkeley, California, United States of America; Medical College of Wisconsin, UNITED STATES OF AMERICA

## Abstract

Spirochetes are a widespread group of bacteria with a distinct morphology. Some spirochetes are important human pathogens that utilize periplasmic flagella to achieve motility and host infection. The motors that drive the rotation of periplasmic flagella have a unique spirochete-specific feature, termed the collar, crucial for the flat-wave morphology and motility of the Lyme disease spirochete *Borrelia burgdorferi*. Here, we deploy cryo-electron tomography and subtomogram averaging to determine high-resolution *in-situ* structures of the *B*. *burgdorferi* flagellar motor. Comparative analysis and molecular modeling of *in-situ* flagellar motor structures from *B*. *burgdorferi* mutants lacking each of the known collar proteins (FlcA, FlcB, FlcC, FlbB, and Bb0236/FlcD) uncover a complex protein network at the base of the collar. Importantly, our data suggest that FlbB forms a novel periplasmic ring around the rotor but also acts as a scaffold supporting collar assembly and subsequent recruitment of stator complexes. The complex protein network based on the FlbB ring effectively bridges the rotor and 16 torque-generating stator complexes in each flagellar motor, thus contributing to the specialized motility and lifestyle of spirochetes in complex environments.

## Author summary

The macromolecular complex responsible for motility in spirochetes is the periplasmic flagellum, which is strikingly different from the external flagellum in *Escherichia coli* and *Salmonella enterica*. Here, we leverage a multidiscipline approach to build a molecular model of the spirochetal flagellar motor in the Lyme disease spirochete *Borrelia burgdorferi*, providing mechanistic insights into spirochete-specific adaptations and their roles in periplasmic flagellar assembly and motility. Knowledge gained from *B*. *burgdorferi* stands to have a broad impact on our understanding of how bacteria evolve species-specific adaptations to thrive in their distinct niches.

## Introduction

Spirochetes are a medically important and ecologically significant group of bacteria with either a distinctive flat-wave or helical morphology [1–6]. Many spirochetes, including *Treponema*, *Borrelia*, and *Leptospira* spp., are highly motile and invasive pathogens responsible for diverse human illnesses, such as syphilis, Lyme disease, and leptospirosis [2,7,8]. Motility is crucial for virulence in all pathogenic spirochetes, and in *B*. *burgdorferi* plays critical roles in each phase of its tick-mouse enzootic life cycle [7,9–13]. The macromolecular complexes that enable spirochetal motility are periplasmic flagella, which are confined to the periplasmic space between the peptidoglycan and outer membrane. Periplasmic flagella are anchored at each cell pole and wrap around the cell cylinder, forming a ribbon and often overlapping in the center of the cell [14–16]. Rotation of the periplasmic flagella exerts force on the cell to generate backward-moving waves along the cell body. This unique mechanism allows spirochetes to penetrate complex host tissue, such as dermal collagen, that inhibits the motility of most bacteria [15–17].

The overall structure of periplasmic flagella, consisting of a motor, hook, and filament, resembles that of external flagella in the model systems *Escherichia coli* and *Salmonella enterica* [18–22]. The motor is a complex, membrane-bound rotary machine powered by the proton (or sodium) gradient across the cytoplasmic membrane. At least 20 different proteins are required to assemble the motor, which can be divided into several morphological domains: the MS and C rings, which are rotor components; the export apparatus, responsible for controlled assembly of downstream structures; the rod, which acts as the flagellar driveshaft; the L and P-rings, thought to serve as bushings at the outer membrane and peptidoglycan layer, respectively; and the stator complexes, which are embedded in the cytoplasmic membrane. Powered by ion motive force, the stator complexes generate torque that drives the rotation of the rotor, rod, hook, and filament [23–25].

While the core components of the flagellum are highly conserved across bacterial taxa [18,20,21], periplasmic flagella are distinct from the external flagella in *E*. *coli* and *S*. *enterica* due to their unique location in the periplasm and spirochete-specific flagellar collar, along with more subtle differences in core structure [10,26–32]. These adaptations are likely important for spirochetes to generate the torque necessary to navigate complex host environments and achieve pathogenesis [19,33,34]. The periplasmic flagellar collar is a large complex ~79 nm in diameter and ~20 nm in height [35]. Five proteins have thus far been determined that directly contribute to the formation of the flagellar collar in *B*. *burgdorferi*: FlcA localizes at the edge of the collar structure and forms part of a turbine-like density in the periphery of the motor, FlcB forms a solenoid structure close to the rod, and FlcC is required for the assembly of a large part of the upper collar structure [35,36]. FlbB and Bb0236 [which we rename here as periplasmic flagellar collar protein D or FlcD] form the base of the collar and are therefore required for assembly of the collar as a whole; cryo-electron tomography (cryo-ET) has revealed that mutants in which either gene is deleted fail to assemble the collar [27,28]. Mutant cell analysis has also established that loss of any of the collar proteins can cause cells to lose their characteristic flat-wave morphology and become rod-shaped, misorient flagella towards the cell pole, and destabilize the stator complexes, rendering them motility deficient [27,28,35,36].

Despite recent progress in characterizing the five flagellar proteins that contribute to the spirochete-specific flagellar collar and stator assembly, many important questions remain: 1) How does the spirochete-specific flagellar collar assemble? 2) How does the flagellar collar recruit, interact with, and regulate the torque-generating stator complexes? and 3) How do the collar proteins function in relation to the rotor of periplasmic flagella? Leveraging *in-situ* motor structures derived from each flagellar collar mutant, protein-protein interactions, and protein structure prediction with AlphaFold2, we map each of the five collar proteins into our *in-situ* motor map, revealing a protein network that bridges the rotor and stator complexes. Importantly, FlbB forms a distinctive ring around the flagellar rotor and serves as the base of collar assembly. Collectively, our findings provide molecular insights into the assembly and adaptations of periplasmic flagella and their impacts on the unique motility of spirochetes.

## Results

### FlbB forms a distinctive periplasmic ring around the MS-ring

**Fig 1:**
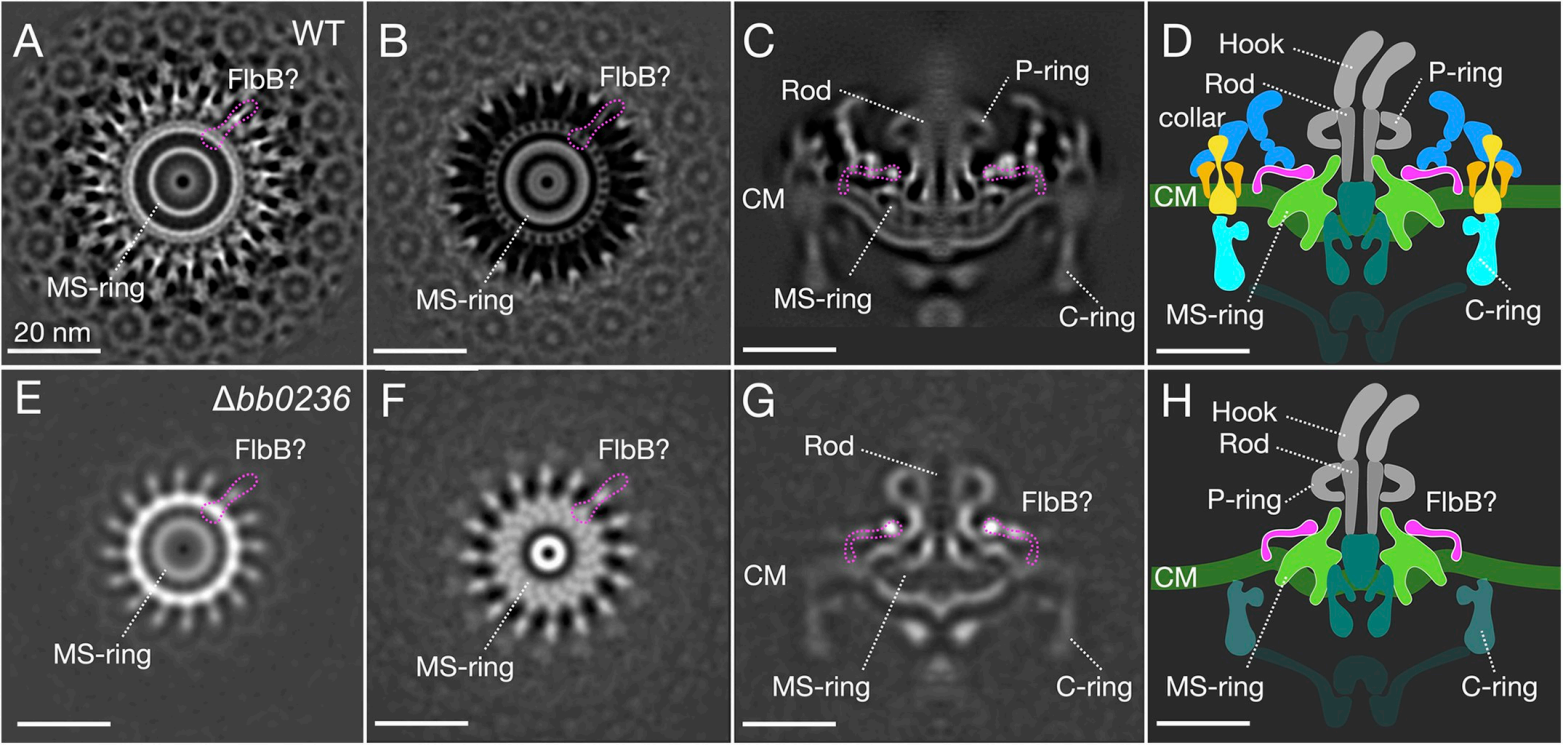
16 spoke-like structures at the base of the flagellar collar form a periplasmic ring around the MS-ring. (**A**, **B**) Two different cross sections of an *in-situ* structure of the wild-type flagellar motor show 16 spoke-like structures, with one subunit outlined in pink around the MS-ring. (**C**) A central section of the wild-type flagellar motor structure shows that the spoke-like structures around the MS-ring are located at the base of the collar. (**D**) A cartoon of the wild-type flagellar motor. Spoke (pink), stator (orange), MS-ring (green), collar (blue), export apparatus (teal), C-ring (light blue). (**E**, **F**) Two different cross sections of an *in-situ* structure of the Δ*flcD/*Δ*bb0236* flagellar motor show 16 spoke-like structures around the MS-ring, with one subunit outlined in pink. (**G**) A central section of the Δ*flcD* flagellar motor structure shows that the spoke-like structures remain associated with the MS-ring, while other collar components are absent. (**H**) A simplified model of the Δ*flcD* flagellar motor. All scale bars 20nm.

To better understand the unique assembly of periplasmic flagella, we determined the *in-situ* structure of the *B*. *burgdorferi* wild-type flagellar motor at ~13Å resolution using cryo-ET and subtomogram averaging of 5,711 motor particles extracted from 950 cell-tip tomograms (Fig 1A–1C and S1 Table). The MS-ring is surrounded by a collar composed of complex, interconnected protein densities. We overcame the complexity of mapping any of the five collar proteins of the flagellar collar structure by systematically examining motor structures derived from new cryo-ET data of the Δ*bb0236* mutant [28] (Fig 1E–1G) and others. For consistency with the other collar proteins, we rename Bb0236 periplasmic flagellar collar protein D (FlcD) and use FlcD and Bb0236 interchangeably. The Δ*bb0236/*Δ*flcD* mutant was an ideal starting point because, as we previously showed, the Δ*flcD* motor structure lacks most of the collar structure, simplifying the task of identifying densities attributable to individual proteins [28]. Our new Δ*flcD* motor structure determined by cryo-ET and subtomogram averaging 858 particles at ~44 Å resolution (S1 Table) reveals 16 spoke-like densities connected to a circular hub surrounding the MS-ring (Fig 1E–1G). The hub-and-spoke structures also appear in the wild-type motor at higher resolution (Fig 1A–1C). Notably, the 16 spoke-like densities connect to a circular hub that appears to have 32-fold symmetry (Fig 1B). We reasoned that FlbB forms the spoke structures because these are present in the Δ*flcD* motor but absent from the Δ*flbB* motor [27,28]. This model is also consistent with our previous finding that a chimeric FlbB-GFP fusion protein is located in a similar region [27].

**Fig 2:**
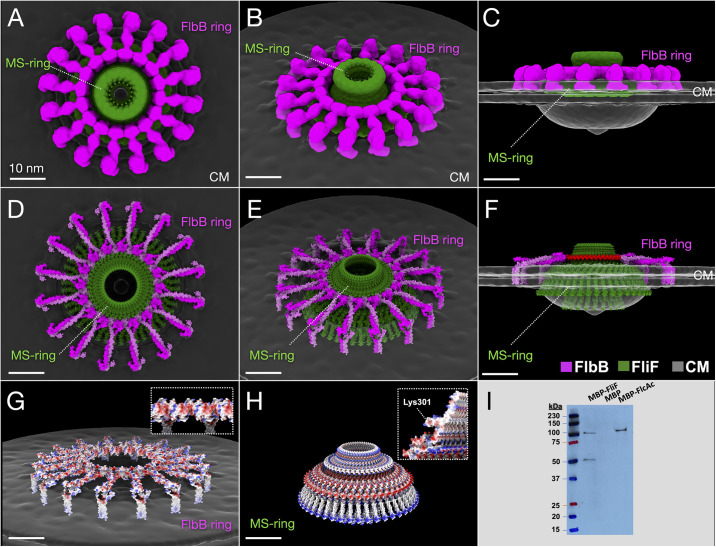
FlbB forms the periplasmic ring around the MS-ring. (**A-C**) Surface views of the FlbB ring around the MS-ring in three directions, respectively. (**D-F**) 3D architecture of the MS-ring and surrounding FlbB ring. (**F**) A side view of the FlbB ring (pink) and MS-ring (green) highlights the loop (red) in FliF that interacts with FlbB. (**G**) The FlbB ring is colored according to electrostatic potential. Negatively charged (red) portions of the structure face the interior of the ring, and positively charged (blue) areas lie on either end of the transmembrane helices. (**H**) The MS-ring is colored according to electrostatic potential. Lys301 is on the end of the protruding loop. (**I**) Pull-down assay shows interaction between FlbB and FliF. FlcAc (C-ter of FlcA) was used as a positive control. The size of the MBP-FliF (and MBP-FlcAc) is 109 kDa. The 50 kDa protein band may be a degradation product from the MBP-FliF.

To explore this model computationally, we built an initial *in-situ* molecular model of FlbB based on our cryo-ET maps of the Δ*flcD* flagellar motor and structures predicted by AlphaFold2 [37]. This initial model was then refined using the high-resolution map of the wild-type motor as a guide. FlbB is a relatively small protein of 205 amino acids (S1 Fig), and the predicted monomer structure consists of an N-terminal transmembrane helix, long ⍺-helical linker region, and C-terminal globular head domain (S2 Fig). As both 16-fold spokes and a 32-fold ring are visible in our *in-situ* structure, and the FlbB-FlbB interaction was confirmed by bacterial two-hybrid assays [27], we reasoned that each spoke is likely formed by a FlbB dimer. The dimer structure predicted with high confidence by AlphaFold Multimer [37] suggested that the long linker regions form a parallel coiled coil (S2 Fig). The coiled coil multimer prediction algorithm LOGICOIL [38] also predicted that FlbB dimerizes as a parallel coiled coil, and the ‘a’ positions of the heptad repeats are well conserved across FlbB homologs, supporting the AlphaFold2 model (Fig 2) and FlbB-FlbB interaction experiment [27].

To further validate the model and to determine the solution structure of FlbB, we performed size-exclusion chromatography coupled with small-angle X-ray scattering (SEC-SAXS) at the SIBYLS Beamline 12.3.1 (Lawrence Berkeley National Laboratory, Berkeley, CA). The prominent elution peak (S3 Fig) derived the final merged SAXS profile (S4A Fig), giving the linear Guinier plot with a radius of gyration (Rg) of 46.5 Å ± 0.9 (S4A Fig inset). The molecular weight calculated from SAXS is 41.5 kDa (see Methods), suggesting a dimer, which is in good agreement with the size of the monomeric FLAG-FlbB-His_6_ protein estimated to be 20.85 kDa. The pair distance distribution function shows multidomain protein with maximal dimension of the macromolecule (Dmax) value ~160 Å (S4B Fig). The normalized Kratky plot also supports the multidomain (dimer) protein with a partially folded form (S4C Fig). The best-fitting AlphaFold3 model fits the SAXS data with a χ^2^ = 2.85 (S4A Fig). The fit was significantly improved when the AlphaFold3 model underwent conformational sampling with BilboMD restrained by PAE and plDDT scores (S5 Fig). The ensemble state model (S4D Fig) fits well with χ^2^ = 0.88 (S4A and S4C Fig). The ensemble fit, which consisted of 2 states at 47% and 53%, suggests high mobility of the N-terminal helix and the C-terminal globular domains (S4D Fig).

Importantly, the C-terminal head domains of the FlbB dimers fit well into the hub density surrounding the MS-ring, the parallel coiled coil region forms the spoke-like structure, and the N-terminal hydrophobic transmembrane helices come just before an unstructured stretch of protein, allowing them to bend 90 degrees from the coiled coil region (Figs 2A–2F and S2 and S1 Movie). The cross-correlation value of the fitting, 0.71, indicates a good match between the density and the model (S2 Table). Together, our *in-situ* structures suggest that sixteen FlbB dimers form the hub structure surrounding the MS-ring, with their C-terminal head domains forming spoke-like structures and N-terminal hydrophobic helices inserted into the cytoplasmic membrane at the base of the collar (Fig 2D–2F). Notably, the model is consistent with our previous findings in which the strain expressing FlbB with a C-terminal GFP tag lacks this ring structure in its flagellar motor [27], as a large C-terminal tag would be expected to disrupt the multimerization of the C-terminal domain of FlbB.

Given the proximity between FlbB and FliF in our model, we tested for direct interaction between FlbB and FliF by pull-down assay and far-western blotting. Both experiments showed specific interactions (Fig 2I). To better understand the potential functions of the FlbB ring and its association with the MS-ring, we built a model of the 46-fold symmetrical *B*. *burgdorferi* MS-ring by flexible fitting the AlphaFold2-predicted structure of *B*. *burgdorferi* FliF into a cryo-ET density map of the *Salmonella enterica* flagellar motor (PDB: 7CGO) [39]. The FlbB ring is modeled in close proximity to an unstructured loop extending from the MS-ring (FliF residues 293–311), with FliF Lys301 at the tip (Fig 2F and 2H). It is worth noting that this loop was not modified from the initial AlphaFold2 model and was predicted with high confidence (S2E and S2F Fig). The MS-ring-facing interior surface of the FlbB ring contains acidic patches contributed by E146, E153, D154, and D188, suggesting potential electrostatic interactions between the two structures (Fig 2G).

### An interconnected protein network spans the radius of the motor

To determine the proteins responsible for other densities in the flagellar collar, we systematically compared newly refined *in-situ* motor structures of previously studied collar mutants with those of wild-type and Δ*flcD* strains [40]. Any density present in the wild-type map but absent from a mutant map lacking a collar protein likely represents the collar protein itself or another protein that depends on it for assembly. Fitting the AlphaFold2-predicted structure of the collar protein into one of these densities would provide further evidence to verify if the density is formed by the protein.

**Fig 3:**
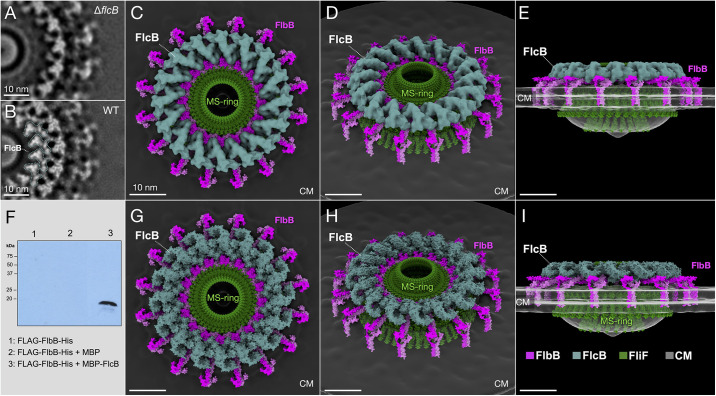
The FlbB ring functions as a template for the assembly of FlcB. (**A** and **B**) Two central slices of the refined *in-situ* structures of the Δ*flcB* and wild-type flagellar motors, respectively. Notably, the densities corresponding to FlcB are absent from the *flcB* mutant. (**C-E**) Three views of the segmented FlcB structure (teal) around the models of the MS-ring (green) and FlbB ring (pink) in the wild-type motor. (**F**) Pull-down assay probed with anti-FLAG Ab shows interactions between FlbB and FlcB. (**G-I**) Three different views of the structures of FlcB in place of the densities above FlbB. All scale bars 10nm.

Comparing the Δ*flcB* motor structure with that of wild type previously revealed that a large, solenoid-shaped structure on top of the FlbB spoke is absent from the mutant motor [35]. We confirmed interaction between FlbB and FlcB by pull-down assay (Fig 3F). Segmentation of our new 24 Å map of the Δ*flcB* motor averaged from 1,693 motors shows that the solenoid structure comprises a 16-fold repeating density, each repeat of which forms one turn of the solenoid (Fig 3C–3E and S1 Table). Sixteen copies of the predicted structure of the tetratricopeptide repeat and circular head domains of FlcB fit into this density with a cross-correlation value of 0.58 (S6A–S6D Fig and S2 Table and S1 Movie), suggesting that FlcB forms the large solenoid-shaped density near the FlbB dimers (Fig 3G–3I). Additionally, the AlphaFold Multimer-predicted structure [41] of the FlbB-FlbB-FlcB trimer matches our model well (S6E and S6F Fig).

**Fig 4:**
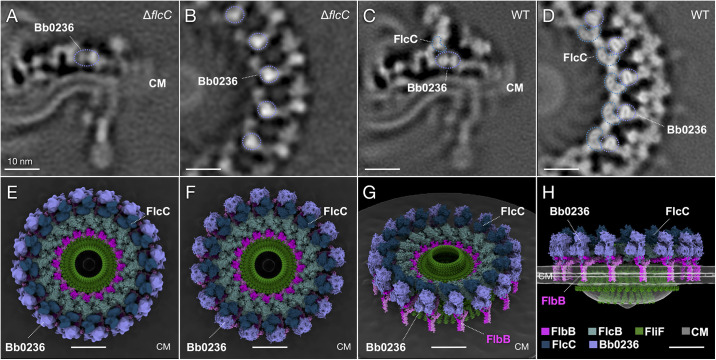
FlcC and Bb0236/FlcD are positioned centrally within the collar. (**A** and **B**) Two perpendicular slices of the focused refined map of the Δ*flcC* flagellar motor. (**C** and **D**) Two perpendicular slices of the refined map of the wild-type flagellar motor. The FlcC structure (dark blue) is missing from the Δ*flcC* mutant, but Bb0236/FlcD (light blue) remains. (**E**) The segmented FlcC and Bb0236/FlcD structures around the models of the MS-ring, FlbB ring, and FlcB in the wild-type motor. (**F-H**) Three different views of the surface-rendered FlcC and Bb0236/FlcD structures in place of the segmented densities. All scale bars 10nm.

We also previously showed that the upper portion of the collar is missing from the Δ*flcC* motor structure [35] (Fig 4A). FlcC, however, is only 320 amino acids long (299 without the predicted Sec signal sequence), and its predicted structure is considerably smaller than the missing density, suggesting that FlcC does not compose this entire upper part of the structure. We therefore hypothesized that other unknown proteins that depend on FlcC for their assembly are likely responsible for the top part of the collar structure.

Our new map of the Δ*flcC* motor was averaged from 2,435 motors at an estimated resolution of 27 Å (S1 Table). As assembly of the collar in the Δ*flcC* motor would stall at the step when FlcC normally assembles, FlcC should form a structure adjacent to but not overlapping with the densities in the Δ*flcC* motor. A density just above FlcB meets these criteria, and we mapped FlcC in this location (Fig 4E–4G). Notably, with only minor adjustments to the relative positions of its two domains, the predicted AlphaFold2 structure of FlcC fits into the density with a cross-correlation value of 0.69, supporting its assignment as FlcC (S2 Table and S7D–S7F and S7G–S7H Fig and S1 Movie). FlcC is also present in 16 copies and, along with another density, forms a continuous ring structure (Fig 4D).

As Bb0236/FlcD is required for the assembly of almost the entire collar, the Δ*flcD* motor does not provide much information to help localize this protein. Given that FlbB is the only collar protein visible in the absence of FlcD and that FlbB also interacts with FlcD [28], FlcD should be adjacent to FlbB within the collar. We propose that Bb0236/FlcD and FlbB form the platform or base of the collar, assemble before FlcB and FlcC, and thus that FlcD should be present even in the absence of FlcB and FlcC. We mapped Bb0236/FlcD to a density in contact with FliL, FlbB, and FlcC, consistent with the importance of Bb0236/FlcD for downstream assembly of the collar and presence in the Δ*flcB* and Δ*flcC* mutant motors (Fig 4). The predicted structure of Bb0236/FlcD fits well into this density, with cross-correlation value 0.63, further supporting the model (S2 Table and S7A–S7C Fig and S1 Movie). Like the other collar proteins, Bb0236/FlcD follows the overall 16-fold symmetry of the *B*. *burgdorferi* periplasmic flagellar collar. The β-propeller domain of Bb0236/FlcD is oriented up toward the parts of the collar whose assembly is dependent on FlcC, suggesting that Bb0236/FlcD may directly interact with the component proteins in addition to supporting their assembly by precipitating the assembly of FlcC (S7A–S7D Fig).

### FlcA interacts with FliL and stabilizes the stator complexes

**Fig 5:**
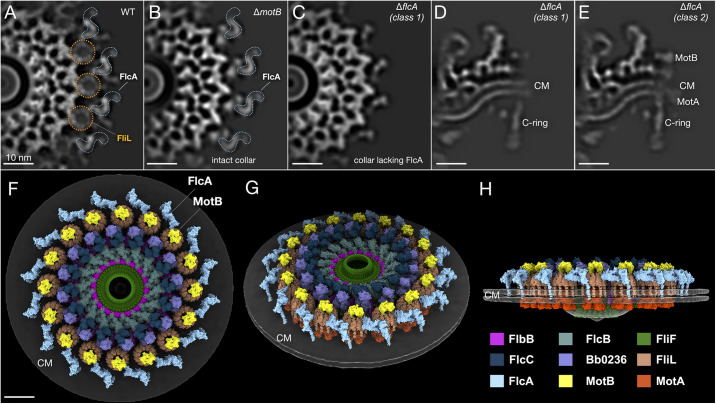
FlcA forms a distinct, S-shaped structure interacting with the stator complex. (**A-C**) Central slices of the refined motor structures from wild type, Δ*motB*, and Δ*flcA* (one class). FlcA (blue) appears to interact with the stator complex through the FliL-ring (orange). (**D** and **E**) Slices of two different class averages from the Δ*flcA* flagellar motors. The stator complex is not visible in one class (**D**) but is visible in the other class (**E**), suggesting that the stator complex is less stable in the absence of FlcA. (**F-H**) The surface-rendered model of the flagellar collar, MS-ring, and stator complexes, including each of the known proteins, in three different views. All scale bars 10nm.

Our previous study provided evidence that the collar mutant Δ*flcA* is non-motile and that both the distal part of the collar and stator complexes are absent from a low-resolution cryo-ET structure, suggesting that FlcA is located at the periphery of the collar and plays a role in recruitment and/or assembly of the FliL ring and stator complex [36]. To better understand the structure and function of FlcA, we determined the *in-situ* structures of the Δ*flcA* motor at 27 Å, a higher resolution than previously published, from 1,286 motors and compared them with the motor structures from wild-type and Δ*motB* cells, revealing an S-shaped density at the periphery of the collar (Fig 5A–5E and S1 Table). The predicted structure of the C-terminal, periplasmic tetratricopeptide repeat domain of FlcA fits nicely into the S-shaped density, with a cross-correlation value of 0.56, and requires only a minor adjustment to properly orient its transmembrane helix, leading us to propose that FlcA is responsible for the 16 S-shaped densities (S2 Table and S8 Fig and S1 Movie). This assignment is consistent with reported interactions between the C-terminal domain of FlcA and FliL and between FlcA and MotB (Figs 5F–5H and S9) [36].

In addition to identifying the density corresponding to FlcA, we obtained the Δ*flcA* motor structures as two distinct classes: one class lacks stator complexes entirely, while the other retains some stator density (Fig 5C–5E). These data suggest that, while FlcA has a function in promoting optimal stator occupancy, it is not essential for stator association with the motor and may be primarily involved in preventing stator disassociation.

### The known collar proteins form only part of a complex network

Our model predicts a network of protein-protein interactions within the *B*. *burgdorferi* collar structure that is far more extensive than previously thought (S9 Fig) [27,28,35,36]. Many of the newly predicted interactions have been tested experimentally by co-immunoprecipitation, pull-down assays, bacterial two-hybrid analysis, and far-western blotting (Figs 2I and 3F). Critically, our model largely agrees with the experimental results, further supporting the positions of these proteins within the motor (S9 Fig and S3 Table).

**Fig 6:**
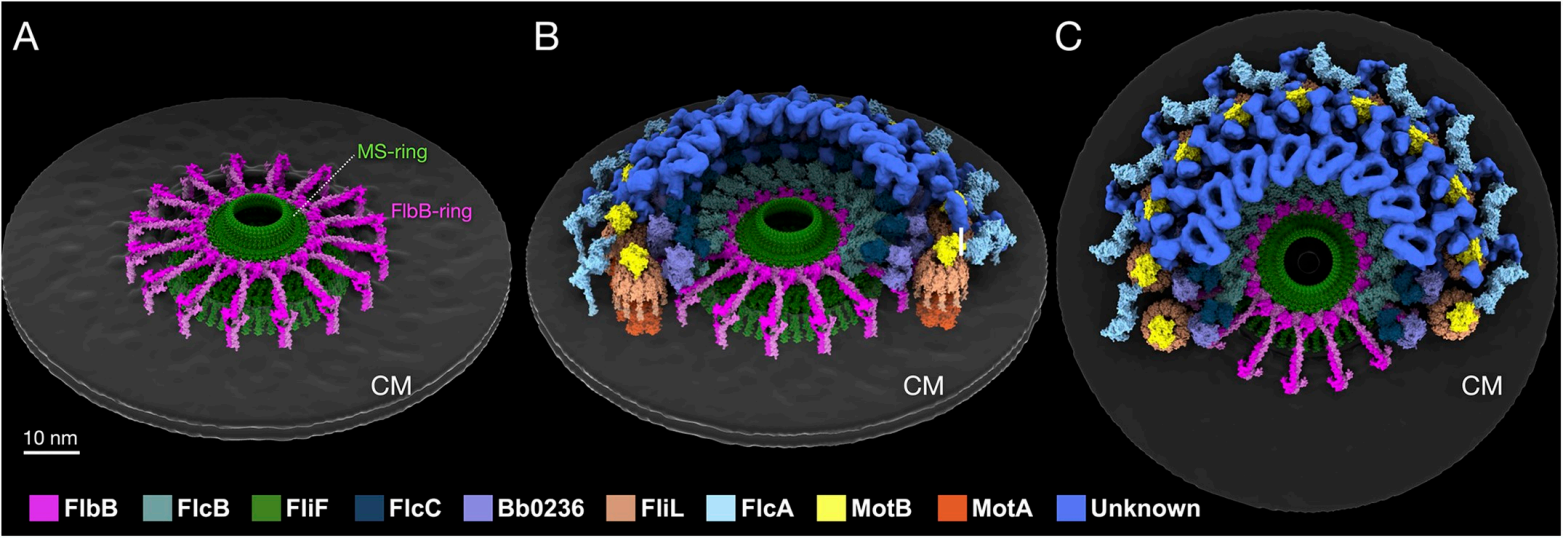
Architecture and assembly of the *B*. *burgdorferi* flagellar motor. (**A**) FlbB interacts with the MS-ring to form the FlbB-ring, likely initiating the assembly of the entire collar. (**B** and **C**) The architecture of the intact flagellar motor, including each known collar protein and the stator complex, is presented in two different views. Notably, the segmented densities corresponding to unknown collar proteins are colored in blue. The model is partially opened to show how the FlbB ring (pink) enables assembly of the intact motor.

Along with the structural information of the known collar proteins, our model reveals large unassigned densities remaining in the flagellar collar (Fig 6B and 6C). The positions of these densities suggest characteristics of the corresponding proteins, such as binding partners and solubility, potentially guiding screens for additional collar proteins. For instance, while the bulk of the unassigned densities compose the upper and outer parts of the collar, one density sits in the middle of FlcB, FlcC, and Bb0236/FlcD and likely belongs to an interaction partner of one or more of these proteins (S9 Fig). This density is also adjacent to an unassigned region that appears to interact with the inner membrane, suggesting that it may belong to a transmembrane or lipidated protein. The protein responsible for this density may be required for the FlcB and FlcC assembly, which is otherwise only directly dependent on Bb0236/FlcD.

## Discussion

Spirochetal motility is highly unusual among flagellated bacteria due to the internal, helix-shaped flagella that rotate to generate backward-moving waves and allow spirochetes to swim [2,42]. As spirochetes are among the most invasive pathogenic bacteria, their lifestyle requires specialized motility machinery to navigate complex host environments [34,43]. The flagellar collar, an adaptation specific to spirochetal motility, plays an important role in both the recruitment of the torque-generating stator and the proper orientation of periplasmic flagella [27,28]. Detailed examination of the structure, composition, and function of the collar is crucial for a deeper understanding of the unique adaptations, motility, and pathogenicity of spirochetes.

### The FlbB ring functions as a scaffold for ordered collar assembly

It is challenging to dissect much of the exact assembly process of the collar. The cryo-ET maps of the Δ*flbB* and Δ*flcD* flagellar motors, in combination with our current segmentation, show that FlbB is capable of localizing to the flagellar motor independently of any other collar component and that FlbB is the only collar component capable of assembling in the absence of Bb0236/FlcD (Fig 1E–1H) [28]. These observations are consistent with a model in which FlbB and Bb0236/FlcD serve as the foundation for collar assembly. Our data do not focus on downstream collar assembly; however, FlcA, FlcB, and FlcC seem to assemble independently. Stator assembly appears to be somewhat dependent on FlcA as the stator units are present in some motors in Δ*flcA*. FlcA, on the other hand, assembles independently of the stator, as the collar protein structure is present in the motors assembled by Δ*motB* cells (Fig 5B and 5E). Detailed analysis of the hierarchical assembly process of the flagellar collar will require further identification of additional collar proteins.

### Interaction between the FlbB ring and MS-ring is likely crucial for flagellar rotation

Our model suggests that electrostatic interactions exist between the FlbB and MS-rings. While the nature and consequences of these interactions remain unexplored, electrostatic interactions between concentric structures within the flagellar motor have also been predicted between the L/P-rings and flagellar rod in *S*. *enterica* [39,44]. The L/P-rings are critical flagellar components in most externally flagellated gram-negative bacteria and create openings in the outer membrane and cell wall, respectively, to allow the flagellar rod to assemble through the periplasm and into the extracellular space. In addition, electrostatic interactions between the negatively charged surface of the rod and rings of charged residues on the interior surfaces of the L-ring are thought to keep the rod centered and balanced, enabling higher rotation speeds and torque transmission with very little friction with the rod. Closer electrostatic interactions between the P-ring and rod are necessary for exporting flagellar proteins into the media, and it is theorized they are necessary for efficient assembly of the P-ring around the rod [44].

According to our model, the FlbB ring is situated within the collar such that it interacts with the MS-ring at an interface defined by the negatively charged residues Glu146, Glu153, Asp154, and Asp188 on the lateral surface of FlbB and Lys301 at the tip of a flexible loop in FliF (Fig 2F–2H). While the function of this interaction would seem to localize FlbB in parallel to the assembly of FlgI/P-ring, it is notable that the FlbB-GFP fusion protein characterized in a previous study localizes to the flagellar motor despite the disruption of the C-terminal domain [27]. This suggests that the interactions between this domain and FliF are not necessary for localization but rather serve a different function. The similarity of the modeled interactions between the P-ring and rod and between the FlbB and MS-rings also implies that the FlbB ring does not rotate with the MS-ring, just as the P-ring does not rotate with the rod (S2 Movie). Given the highly interconnected nature of the flagellar collar, the interface between the FlbB and MS-rings appears to be the most likely boundary between the rotating and static parts of the motor, suggesting that the FlbB ring may serve as a bearing enabling stable rotation of the rotor. Dissecting the interaction between FlbB and FliF would help determine which regions of the proteins interact directly and whether the interaction depends on the charged residues identified here. A direct interaction between the flagellar rotor and a presumably stationary part of the highly interconnected stator scaffold structure clearly warrants further investigation.

Interestingly, our model suggests that, in the mature flagellar collar, the FlbB dimers are not adjacent to some published experimental interaction partners, namely FlcA and FlcC [27,35,36] (S9 Fig and S3 Table). Given how well FlbB fits the spoke densities adjacent to the MS-ring and the previously published structure of FlbB-GFP assembled into the motor at roughly the same position [27], our assignment of FlbB to that position seems reasonable. Furthermore, there is simply no position within the motor where a protein the size of FlbB could simultaneously interact with FliF and FlcA, nor are there any membrane-proximal densities remaining unassigned into which FlbB fits. Together, these observations suggest that FlbB has another function that involves interacting with and disassociating from several flagellar collar proteins before the collar structure is fully assembled.

In summary, this study leveraged improved cryo-ET maps and AlphaFold2 to build a molecular model of the *B*. *burgdorferi* flagellar collar. The collar model illustrates a novel hub-and-spoke structure within the collar, with the FlbB ring likely playing a role as a bearing enabling stable rotation of the MS-ring. Importantly, the collar structure acts as a foundation for the assembly of the spirochete-specific stator scaffold, which in turn promotes spirochetal motility. Of the 19 genera of spirochetes with representative genomes deposited in the JGI IMG database, 17 have FlbB homologs of the *B*. *burgdorferi* protein identified by BLAST (S1 Fig). The remaining two genera are in the family Sphaerochaetaceae, whose unique member species are coccoid and non-motile [45]. As FlbB is conserved in other flagellated spirochetes, we expect the FlbB ring is also present in those species. Additionally, our model defines a network of proteins extending from the flagellar rotor to the stator units and identifies elements key to the collar’s stator recruitment function. Beyond the general location of each collar component, modeling AlphaFold2 structures with multiple improved density maps for guidance allowed us to determine specific densities corresponding to each protein and to predict interactions between them. As homologs of each known *B*. *burgdorferi* collar gene exist in other genera of spirochetes, including *Treponema* and *Leptospira*, our findings are broadly applicable across spirochetes.

## Materials and methods

### Bacterial strains and growth conditions

The high-passage, avirulent *B*. *burgdorferi* strain B31-A was used as the wild-type clone throughout the study [46]. All *B*. *burgdorferi* mutants described here were constructed in the B31-A background and reported previously [27,28,35,36].

### Cryo-ET data collection and tomogram reconstruction

Frozen-hydrated specimens were prepared as described previously [26]. In brief, various clones of exponentially growing *B*. *burgdorferi* cells were centrifuged individually at 5,000 ×g for ~5 min, and the resulting pellets were suspended in PBS to achieve a cell concentration of ~1 × 10^8^/ml. After adding a 10-nm gold marker solution, 5μl of the cell suspension was placed on freshly glow-discharged (for ~25 s) holey carbon grids (Quantifoil Cu R2/1, 200 mesh). The grids were front blotted with Whatman filter paper and rapidly frozen in liquid ethane, using a homemade plunger apparatus as described previously [26]. The grids were then imaged using a 300-kV Titan Krios electron microscope (Thermo Fisher Scientific) equipped with a field emission gun and a post-Gatan imaging filter (GIF) K3 Summit direct electron detector (Gatan). SerialEM was used to collect all tilt series at magnification ×42000, corresponding to 2.1 Å /pixel at the specimen level [47]. The defocus was set to -4.8μm. A total dose of ~80 e^-^/Å^2^ is distributed among 33 tilt images covering angles from -48° to 48° with a tilt step of 3°.

All recorded images were first motion corrected using MotionCorr2 [48] and then stacked and aligned by IMOD [49]. Gctf [50] was used to determine the defocus of each tilt image in the aligned stacks, and the “ctfphaseflip” function in IMOD was used to do the contrast transfer function (CTF) correction for the tilt images. The aligned tilt series were used to reconstruct tomograms by simultaneous iterative reconstruction technique (SIRT) reconstruction using Tomo3D [51]. The number of tomograms used in this work for each strain is shown in S1 Table.

### Subtomogram analysis

Bacterial flagellar motors were manually picked from the 8× binned SIRT tomograms. The subtomograms of the corresponding flagellar motors were extracted from unbinned tomograms generated by weighted back-projection (WBP) using IMOD. Subsequently, we generated 4× and 2× binned subtomograms for the following analyses. I3 tomography package [52,53] was used for initial subtomogram analysis of 4× binned data from each strain independently as described previously [35,36]. After several cycles of subtomogram alignment and classification, we determined the 4× binned structures from each mutant, respectively. We then used 2× binned subtomograms to determine the *in-situ* structures of the whole flagellar motor from each mutant, respectively.

### Focused refinement of collar region

Each flagellar motor has 16 collar subunits. After alignment for the whole motor structure, the regions around 16 collar subunits were first extracted from each motor by 16-fold symmetry expansion, and then we refined the 3D alignment and applied 3D classification based on the density of the collar subunit to remove particles with bad contrast or large distortions to obtain the refined structures. The number of flagellar motor and collar subunits used for subtomogram averaging are shown in S1 Table. The resolution of the resulting collar structure from each mutant estimated by Fourier shell correlation (FSC) was presented in S1 Table.

### 3D visualization and modeling

UCSF ChimeraX v. 1.6.1, developed by the Resource for Biocomputing, Visualization, and Informatics at the University of California, San Francisco, with support from National Institutes of Health R01-GM129325 and the Office of Cyber Infrastructure and Computational Biology, National Institute of Allergy and Infectious Diseases [54,55] was used for visualization and surface rendering of subtomogram averages. ColabFold was used to predict secondary and tertiary structures for each collar protein [37], and a local installation of AlphaFold Multimer was used to predict structures of protein multimers [41]. Plots of predicted error values for multimer models were generated using AlphaPickle [56]. Initial fitting of the atomic models into density maps was done using the “fitmap” command in ChimeraX. Density maps were watershed segmented using the Segger [57,58] implementation within ChimeraX, and fits were refined based on that segmentation. Subsequently, manual modifications were applied to the models using Coot [59]. The cross-correlation values between our models and density maps are presented in S2 Table. Symmetry was then applied to each component to construct the comprehensive architecture of the flagellar motor. The entire architecture underwent further optimization through geometry minimization using the Phenix [60] suite.

### Size exclusion chromatography coupled with small-angle X-ray scattering (SEC-SAXS)

Purified recombinant FLAG-FlbB-His_6_ (deleted the first 42 aa from the FlbB) protein was dialyzed using SnakeSkin Dialysis tubing 3500 MWCO (Thermo Fisher Scientifc) in HEPES buffer (10 mM HEPES and 140 mM NaCl, pH 7.3) at 4°C under constant stirring. After dialysis, the protein sample was concentrated to 4 mg/ml by using AmiconUltra -15 Ultracel-3K (Merck Millipore Ltd) centrifugal filter at 5,000 rpm for 20 min at 4°C and sent to the SIBYLS beamline 12.3.1 at Advanced Light Source (ALS) at Lawrence Berkeley National Laboratory [61,62]. We set the X-ray wavelength (λ) to 1.24 Å and the sample-to-detector distance to 2,100 mm. This combination gives scattering vectors (q) ranging from 0.01 Å^-1^ to 0.4 Å^-1^. The scattering vector is q = 4πsinθ/λ, where 2θ is the scattering angle. We coupled the SAXS flow cell to an Agilent 1260 Infinity HPLC system using a Shodex 802.5 SEC column equilibrated with the running buffer as indicated above with a flow rate of 0.65 ml min-1. The eluent was subject to the SAXS measurements, and 2 sec X-ray exposures were collected continuously during a 25 min elution. All frames for analyses had one SAXS frame corresponding to the running buffer before the detection of a peak subtracted from each. The radius of gyration (Rg) was calculated for each of the subtracted frames using the Guinier approximation: I(q) = I(0) exp(−q^2^Rg^2/3^) with the limits qRg < 1.3. The elution peak was compared to the integral of ratios to background and Rg relative to the recorded frame using the program RAW [63]. Uniform Rg values across an elution peak represent a homogeneous sample. Final merged SAXS profiles, derived by integrating multiple frames at the elution peak, were used for further analyses. We calculated the Guinier plot to provide information on the aggregation state, the volume of correlation (Vc) to estimate the molecular weight [64], and the pair distribution function [P(r)] to calculate the maximal inter-particle dimension [65].

### SAXS model fitting

The FlbB-6His dimer was generated from the sequence using AlphaFold3 [66]. The five generated models fit the SAXS using FoXS [67,68]. The best fitting model achieved χ^2^ = 2.85. The predicted aligned error (PAE) matrix, together with the predicted local distance difference test (pLDDT) scores of the top Alphafold3 model, were used to restrain the conformational sampling in conformational fitting by BilboMD [69]. Residues 26–91 of each monomer were fixed, while residues within 1–23 and 93–171 were allowed to move as rigid bodies, which were determined from Leiden clustering of regions of residues with low pLDDT (< 50). An ensemble fit of 2 states achieved the best fit to the SAXS with (χ^2^ = 0.88).

### Pull-down assays

Biophysical interactions between two recombinant proteins are determined as follows. *B*. *burgdorferi* gene coding for FlbB, FlcB, or FliF was cloned in expression vectors containing either 6xHis, such as pET28 from Invitrogen Inc., or MBP, such as pMALc-5x from New England Biolabs (NEB). Specifically, the FlbB was cloned in the pET28 vector to express FLAG-FlbB-His_6_, and FlcB and FliF were cloned separately in the pMALc-5x vector to express MBP-FlcB and MBP-FliF, respectively. Subsequently, the vectors were transformed independently into the *E*. *coli* codon plus cells. The *E*. *coli* cells harboring the expression plasmids were induced separately per the manufacturer’s instructions. The expressed cells were mixed (~1:1 such as FLAG-FlbB-His_6_ and MBP-FlcB or MBP-FliF), french pressed to lyse the bacteria, and purified using amylose or Ni-NTA agarose resin. The purified proteins were subjected to SDS-PAGE and then transferred to a PVDF membrane for immunoblotting with commercially available 6x-His Tag Monoclonal Antibody (for the amylose resin purified proteins) or Anti-MBP Monoclonal Antibody or Monoclonal ANTI-FLAG M2 antibody (for the Ni-NTA agarose resin purified proteins), as per the manufacturer’s instructions. Specifically, the FLAG-FlbB-His_6_ and MBP-FliF mixed proteins were purified using Ni-NTA agarose resin and then the PVDF membrane was probed with Anti-MBP antibody. On the other hand, the FLAG-FlbB-His_6_ and MBP-FlcB mixed proteins were purified using the amylose resin and then the PVDF membrane was probed with Anti-FLAG antibody. The pull-down experiments were conducted at least twice to ensure scientific rigor and reproducibility. The 6x-His Tag, Anti-MBP, and Anti-FLAG antibodies were purchased from Invitrogen, NEB, and Millipore Sigma.

### Far-western blotting

Protein-protein interactions were determined using Far-western or affinity blotting [70] [71]. Briefly, 1 μg of purified recombinant MBP-FliF, MBP-FlcAc (C-terminus of FlcA), and MBP-tagged protein was subjected to SDS-PAGE and coomassie blue staining or transferred to polyvinylidene difluoride (PDVF) membranes. The membranes were blocked in blocking solution (5% skim milk, 0.9% NaCl, 10 mM Tris, pH 7.4) with gentle shaking for 4–6 hours at room temperature and then incubated with purified FLAG-FlbB-His_6_ at a concentration of 2 μg/ml in blocking solution overnight at 4°C. The membranes were washed 3 times with the washing buffer (10mM Tris, 150mM NaCl, and 0.3% Tween 20, pH 7.4) and then probed with a rabbit polyclonal FlbB antibody [27]. The far-western blotting experiments were performed independently twice to ensure scientific rigor and reproducibility.

## Supporting information

S1 Fig FlbB is conserved across flagellated spirochetes. A multi-sequence alignment of FlbB amino acid sequences from each of the flagellated genera of spirochetes. The “a” and “d” positions in the heptad repeats of the predicted coiled coil domain are labeled.
(TIF)

S2 Fig FlbB fits into the spoke density. (**A**, **B**) Watershed-segmented densities from the spoke structures revealed by cryo-ET. The ribbon model of the FlbB dimer has been fit into the density. (**C**) Ribbon model of the predicted structure of the FlbB dimer colored according to the pLDDT confidence score returned by AlphaFold2. (**D**) Overlay between the originally predicted structure of the FlbB dimer (orange) and the final fitted model (purple). Inset is the plot of the Predicted Aligned Error (PAE) returned by AlphaFold Multimer for the FlbB homodimer. (**E**) Ribbon model of the AlphaFold Multimer-predicted interaction between FlbB and the MS-ring protein FliF. Inset is the plot of the PAE returned by AlphaFold Multimer for the FlbB-FliF heterotrimer. Dark blue at point (x, y) represents high confidence in the predicted relative position of residues x and y. Yellow represents low confidence. (**F**) Plot of the PAE returned by AlphaFold Multimer for the FlbB-FlbB dimer.
(TIF)

S3 Fig SEC-SAXS chromatogram. Green bar indicates the data frames used for the analysis of the peak.
(TIF)

S4 Fig Small-angle X-ray (SAXS) scattering results and modeling fitting of FlbB. (**A**) Experimental data (black), the model fits, and fit-residuals of the Alphafold3 structure (orange) and 2-state model (violet). (**B**) Pair distance distribution functions and (**C**) normalized Kratky plots from the experimental SAXS and the model fit curves colored as in the panel **A**. Dashed vertical and horizontal lines correspond to qRg = sqrt(3) and (qRg)^2^(I(q)/I(0)) = 1/e indicating the position of the peak for well-folded protein (**D**) Structures of the Alphafold3 prediction, colored according to pLDDT, and the overlayed BilboMD ensemble states.
(TIF)

S5 Fig Clustering basis for FlbB dimer SAXS modeling. (**A**) Predicted aligned error (PAE) matrix. (**B**) Alphafold3 structure, colored according to PAE clusters. Red boxes on the matrix and model indicate regions in the helix that indicate higher confidence of interaction to each other.
(TIF)

S6 Fig FlcB fits into the solenoid density above FlbB. (**A**-**C**) Watershed-segmented densities from the solenoid structure in the wild-type flagellar motor density map. The FlcB model fits well into the density. (**D**) Ribbon model of the predicted structure of FlcB colored according to the pLDDT confidence score returned by AlphaFold2. (**E**) Ribbon model of the AlphaFold Multimer-predicted interaction between FlbB and FlcB. (**F**) Plot of the PAE returned by AlphaFold Multimer for the FlbB-FliF heterotrimer.
(TIF)

S7 Fig Bb0236/FlcD and FlcC fit into densities near the center of the collar. (**A**-**C**) The predicted model of Bb0236/FlcD fits well in to the watershed-segmented density from the central collar region of the wild-type flagellar motor map. (**D**) The predicted structure of Bb0236/FlcD is colored according to the pLDDT confidence score returned by AlphaFold2. (**E**, **F**) The predicted model of FlcC fits well into the watershed-segmented density from the central collar region of the wild-type flagellar motor map. (**G**) The predicted structure of FlcC is colored according to the pLDDT confidence score returned by AlphaFold2. (**H**) Overlay between the originally predicted structure of FlcC (white) and the final fitted model structure (dark blue).
(TIF)

S8 Fig FlcA fits into a density at the edge of the collar. (**A**-**C**) Watershed-segmented densities from the periphery of the collar in the wild-type flagellar motor density map. The ribbon model of FlcA fits the density. (**D**) Ribbon model of the predicted structure of FlcA colored according to the pLDDT confidence score returned by AlphaFold2. (**E**) Overlay between the originally predicted structure of FlcA (white) and the final fitted model structure (light blue).
(TIF)

S9 Fig A network of protein-protein interactions in the *Borrelia* flagellar collar. Surface-rendered model of the collar is shown in the top panel. A protein-protein interaction network based on our model and existing experimental data is shown in the bottom panel. Notably, most interactions predicted here are consistent with the experimental data (green). Two experimental interactions are not in agreement with the model (red).
(TIF)

S1 Movie This movie shows how we built the model of the spirochetal flagellar motor based on *in-situ* and AlphaFold2-predicted flagellar motor structures. (MP4)

S2 Movie This movie shows how the collar assembles and functions as the key component of the spirochetal flagellar motor. (MP4)

S1 Table Cryo-ET data from wild type and mutants. (DOCX)

S2 Table Fitting AlphaFold2-predicted models into cryo-ET maps. (DOCX)

S3 Table Interactions among collar and stator proteins confirmed experimentally. (DOCX)
