# Supplementary figures and images for: FlbB forms a distinctive ring essential for periplasmic flagellar assembly and motility in Borrelia burgdorferi
Source: PLoS Pathog. 2025 Jan 8;21(1):e1012812. doi: 10.1371/journal.ppat.1012812 (PMC11750108; doi:10.1371/journal.ppat.1012812)

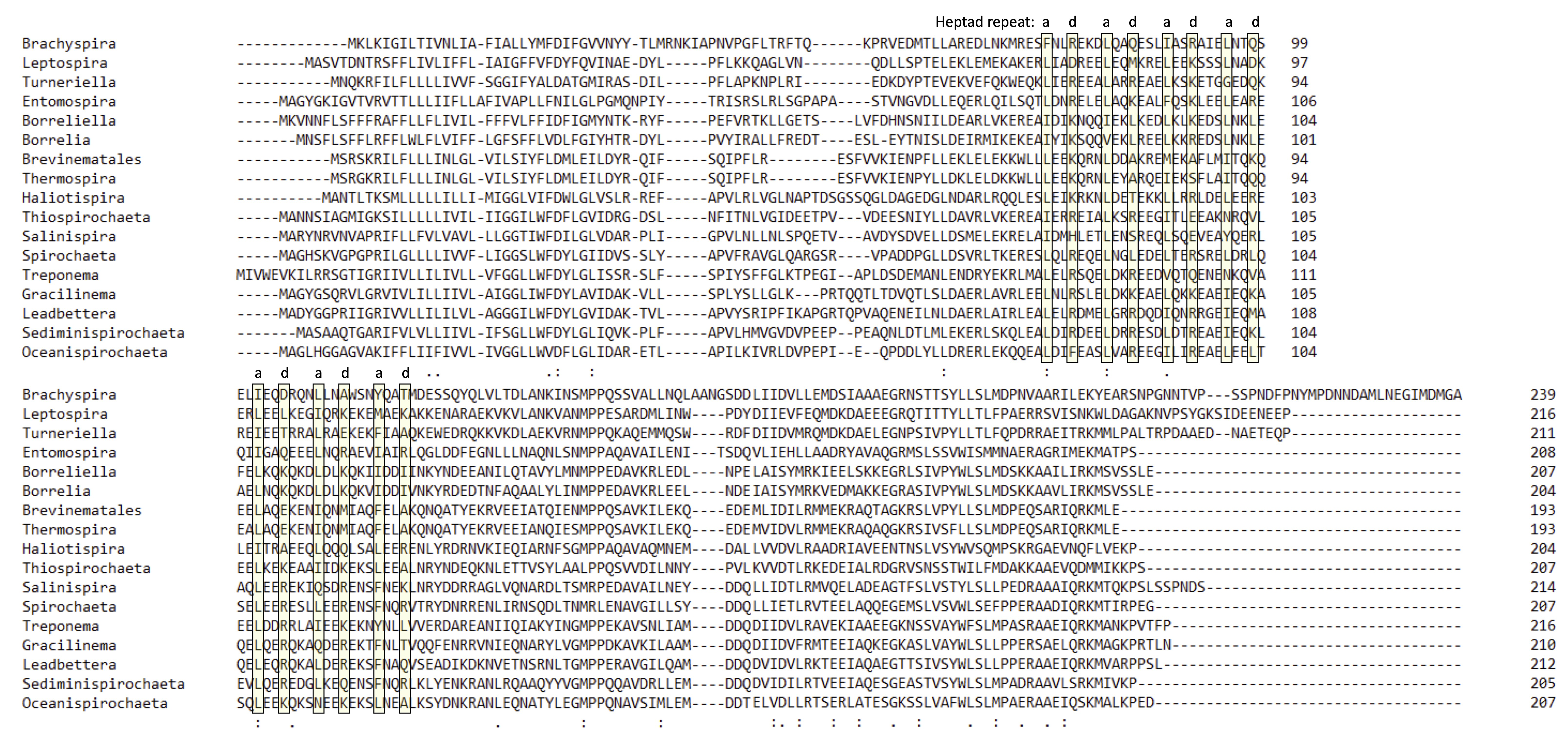

Supplement: S1 Fig — A multi-sequence alignment of FlbB amino acid sequences from each of the flagellated genera of spirochetes. The “a” and “d” positions in the heptad repeats of the predicted coiled coil domain are labeled. (TIF) [file ppat.1012812.s001.tif]

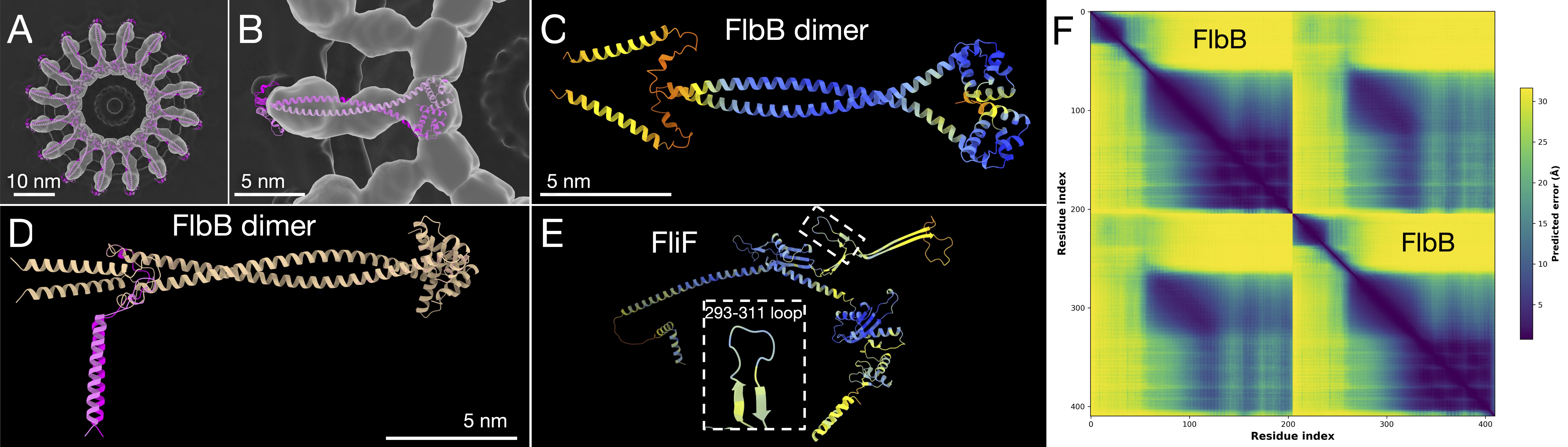

Supplement: S2 Fig — (A, B) Watershed-segmented densities from the spoke structures revealed by cryo-ET. The ribbon model of the FlbB dimer has been fit into the density. (C) Ribbon model of the predicted structure of the FlbB dimer colored according to the pLDDT confidence score returned by AlphaFold2. (D) Overlay between the originally predicted structure of the FlbB dimer (orange) and the final fitted model (purple). Inset is the plot of the Predicted Aligned Error (PAE) returned by AlphaFold Multimer for the FlbB homodimer. (E) Ribbon model of the AlphaFold Multimer-predicted interaction between FlbB and the MS-ring protein FliF. Inset is the plot of the PAE returned by AlphaFold Multimer for the FlbB-FliF heterotrimer. Dark blue at point (x, y) represents high confidence in the predicted relative position of residues x and y. Yellow represents low confidence. (F) Plot of the PAE returned by AlphaFold Multimer for the FlbB-FlbB dimer. (TIF) [file ppat.1012812.s002.tif]

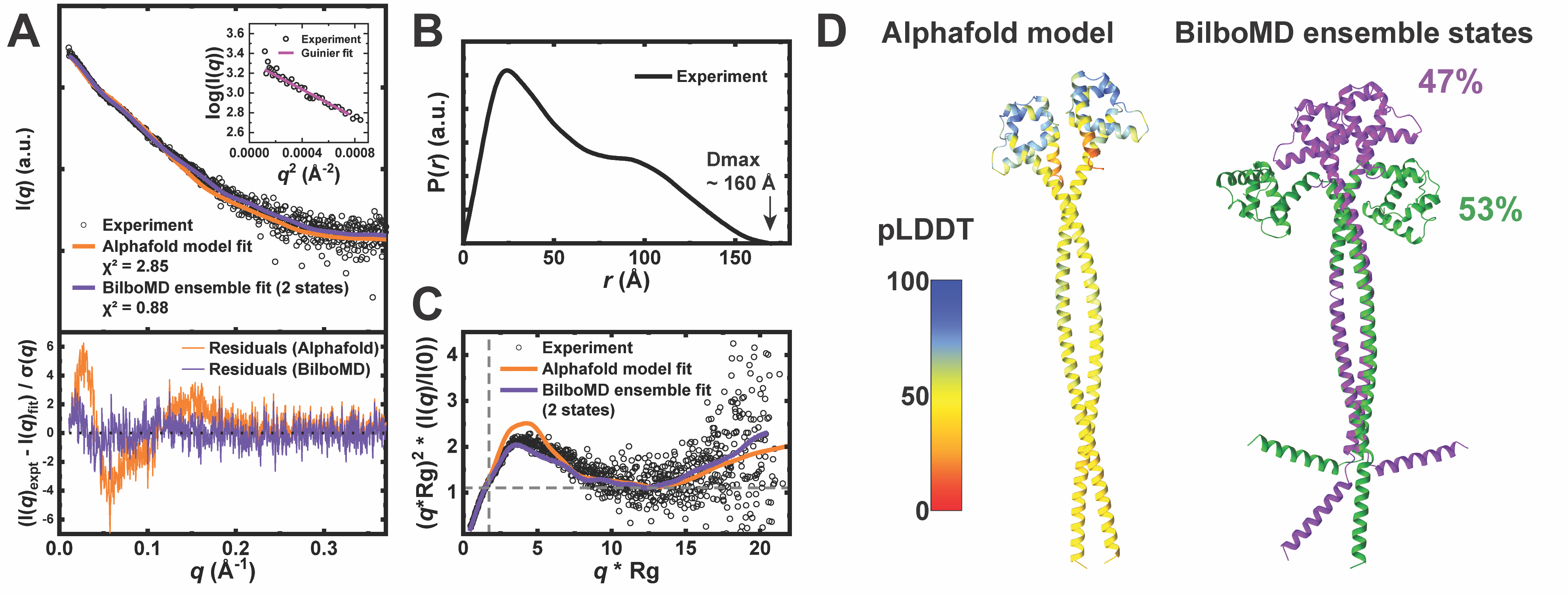

Supplement: S4 Fig — (A) Experimental data (black), the model fits, and fit-residuals of the Alphafold3 structure (orange) and 2-state model (violet). (B) Pair distance distribution functions and (C) normalized Kratky plots from the experimental SAXS and the model fit curves colored as in the panel A. Dashed vertical and horizontal lines correspond to qRg = sqrt(3) and (qRg)2(I(q)/I(0)) = 1/e indicating the position of the peak for well-folded protein (D) Structures of the Alphafold3 prediction, colored according to pLDDT, and the overlayed BilboMD ensemble states. (TIF) [file ppat.1012812.s004.tif]

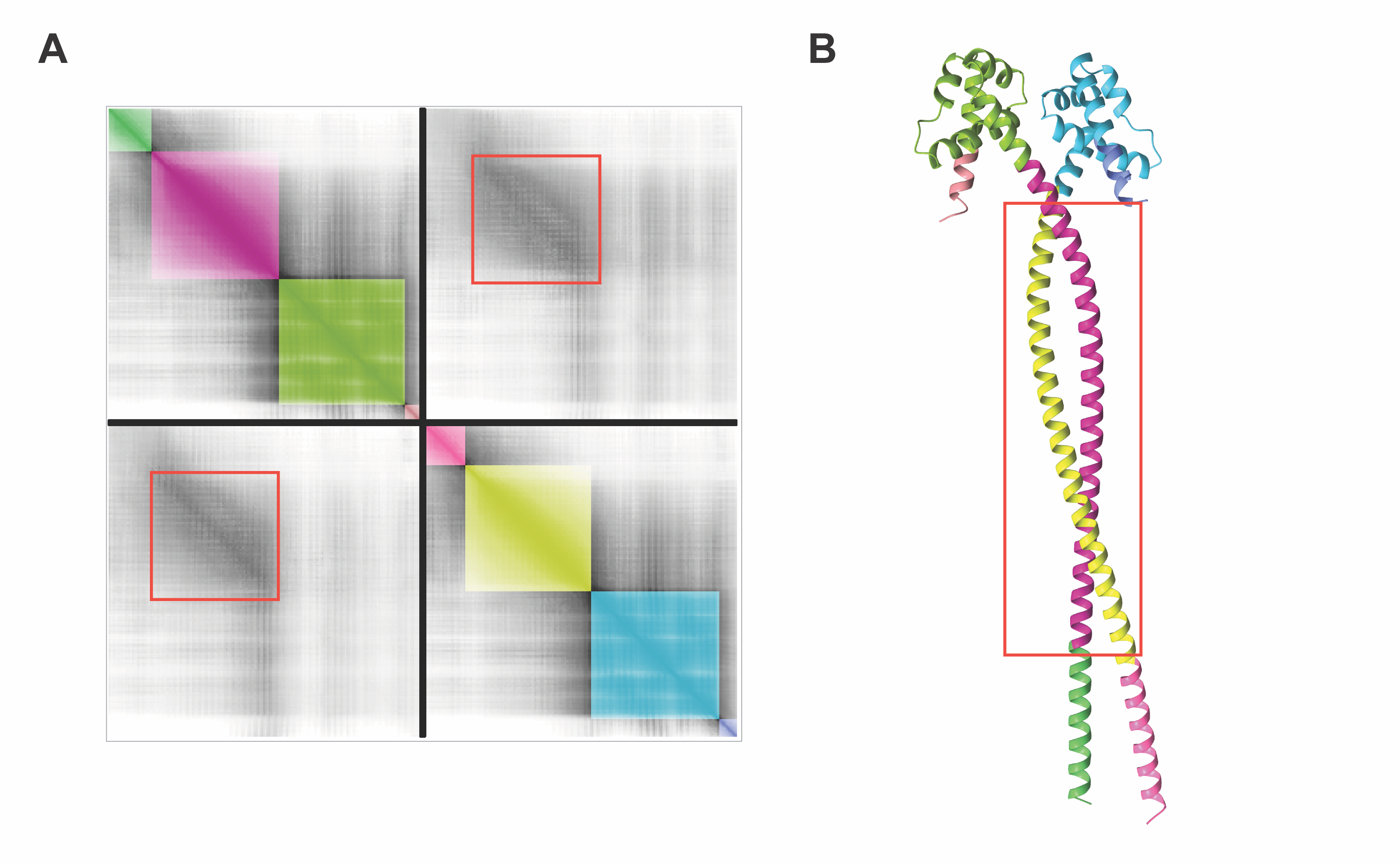

Supplement: S5 Fig — (A) Predicted aligned error (PAE) matrix. (B) Alphafold3 structure, colored according to PAE clusters. Red boxes on the matrix and model indicate regions in the helix that indicate higher confidence of interaction to each other. (TIF) [file ppat.1012812.s005.tif]

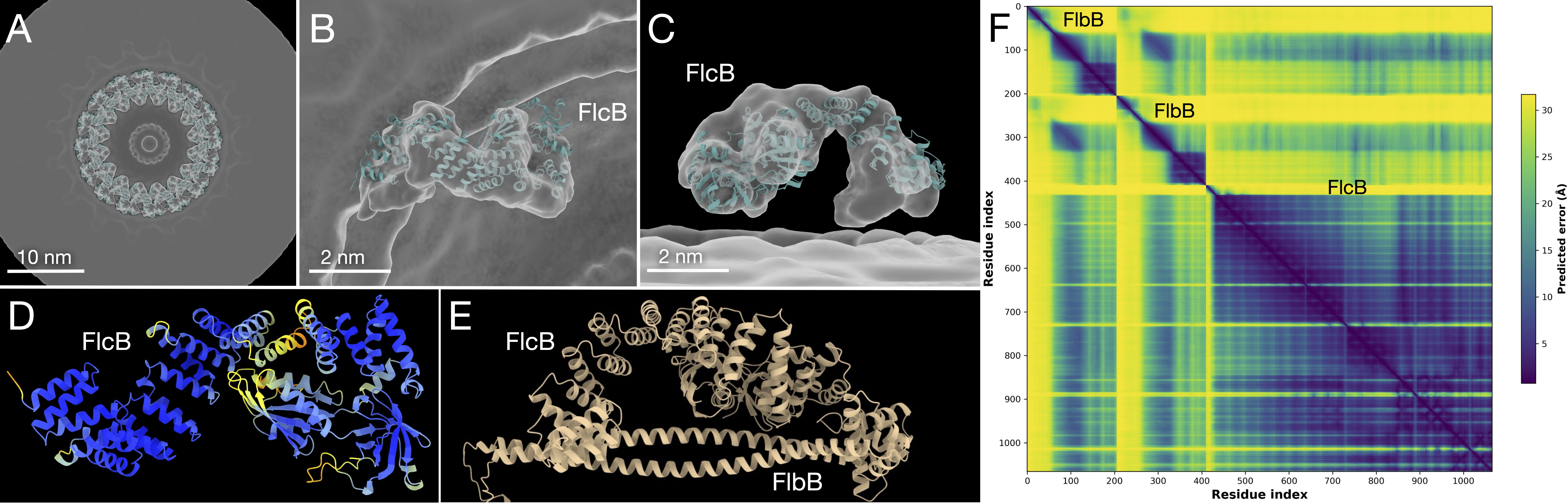

Supplement: S6 Fig — (A-C) Watershed-segmented densities from the solenoid structure in the wild-type flagellar motor density map. The FlcB model fits well into the density. (D) Ribbon model of the predicted structure of FlcB colored according to the pLDDT confidence score returned by AlphaFold2. (E) Ribbon model of the AlphaFold Multimer-predicted interaction between FlbB and FlcB. (F) Plot of the PAE returned by AlphaFold Multimer for the FlbB-FliF heterotrimer. (TIF) [file ppat.1012812.s006.tif]

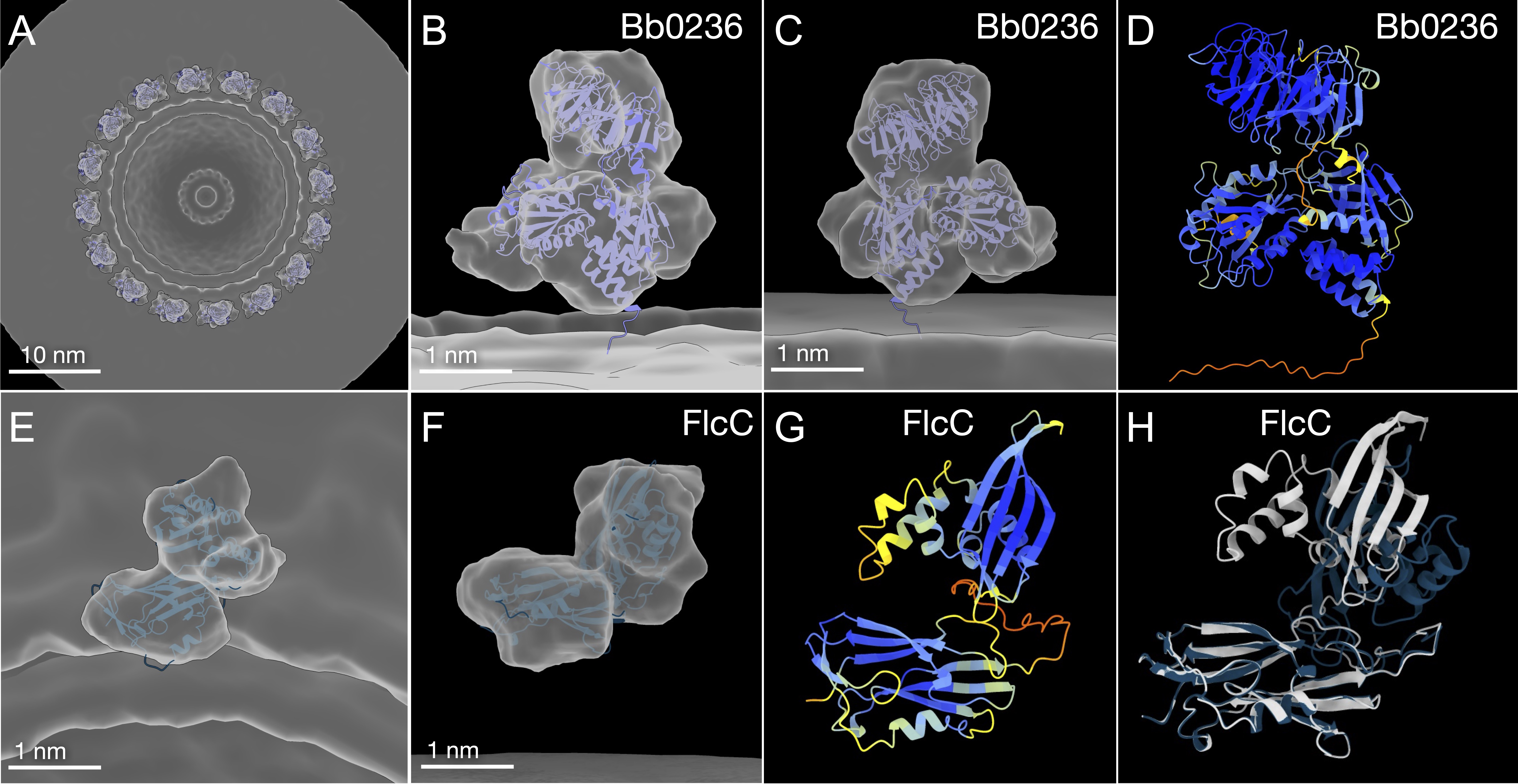

Supplement: S7 Fig — (A-C) The predicted model of Bb0236/FlcD fits well in to the watershed-segmented density from the central collar region of the wild-type flagellar motor map. (D) The predicted structure of Bb0236/FlcD is colored according to the pLDDT confidence score returned by AlphaFold2. (E, F) The predicted model of FlcC fits well into the watershed-segmented density from the central collar region of the wild-type flagellar motor map. (G) The predicted structure of FlcC is colored according to the pLDDT confidence score returned by AlphaFold2. (H) Overlay between the originally predicted structure of FlcC (white) and the final fitted model structure (dark blue). (TIF) [file ppat.1012812.s007.tif]

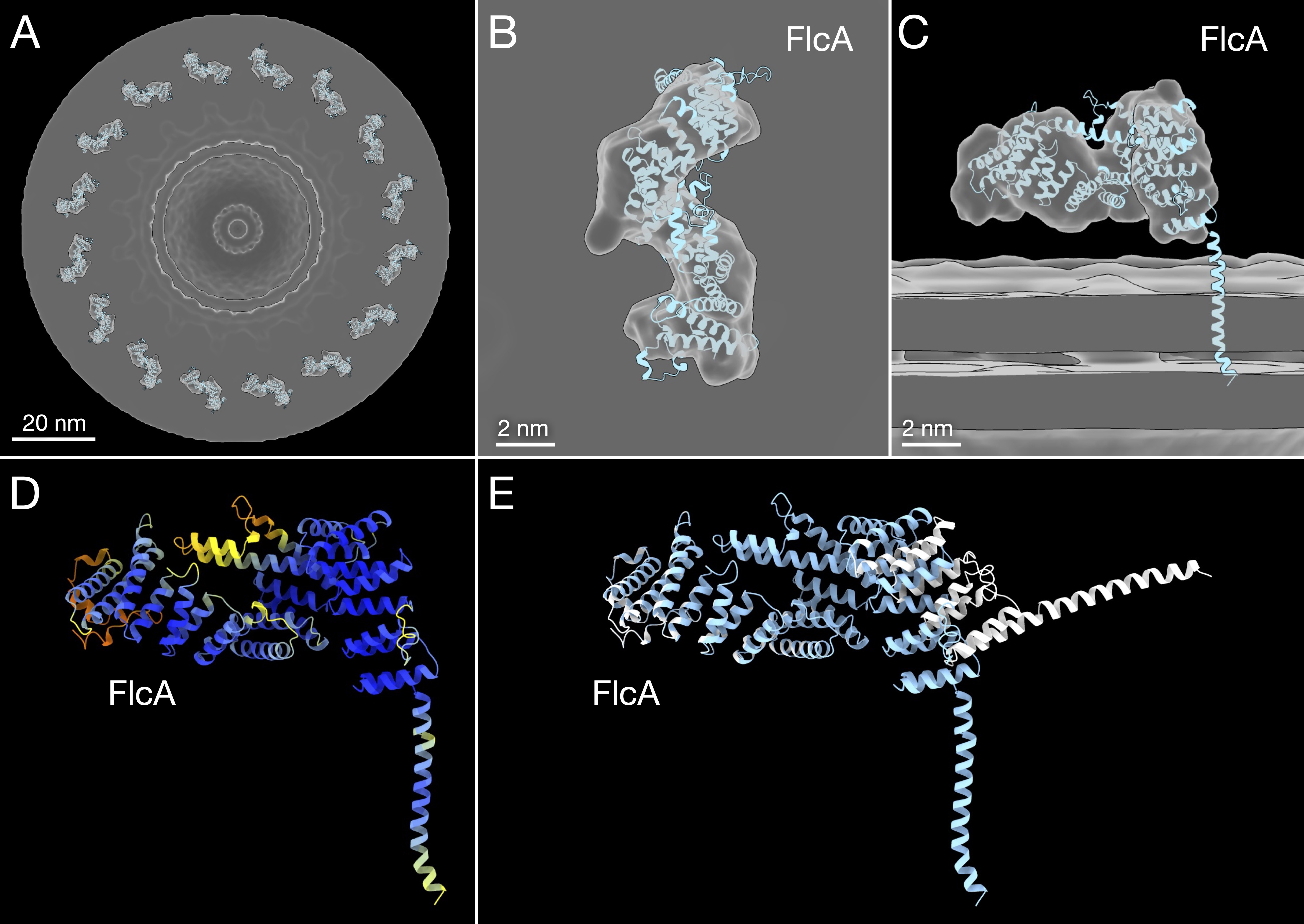

Supplement: S8 Fig — (A-C) Watershed-segmented densities from the periphery of the collar in the wild-type flagellar motor density map. The ribbon model of FlcA fits the density. (D) Ribbon model of the predicted structure of FlcA colored according to the pLDDT confidence score returned by AlphaFold2. (E) Overlay between the originally predicted structure of FlcA (white) and the final fitted model structure (light blue). (TIF) [file ppat.1012812.s008.tif]

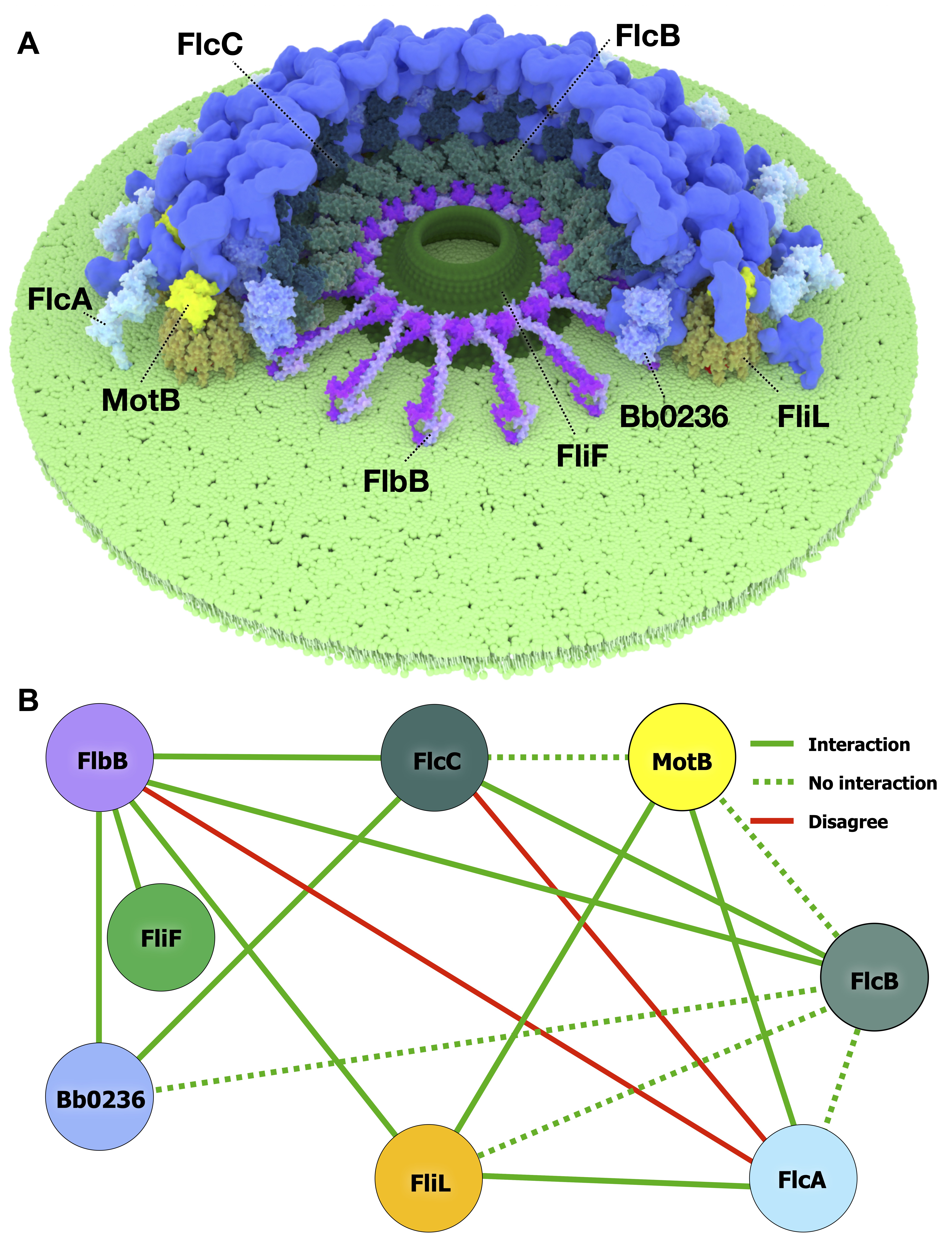

Supplement: S9 Fig — Surface-rendered model of the collar is shown in the top panel. A protein-protein interaction network based on our model and existing experimental data is shown in the bottom panel. Notably, most interactions predicted here are consistent with the experimental data (green). Two experimental interactions are not in agreement with the model (red). (TIF) [file ppat.1012812.s009.tif]
