# Supplementary material for: FlbB forms a distinctive ring essential for periplasmic flagellar assembly and motility in Borrelia burgdorferi
Source: PLoS Pathog. 2025 Jan 8;21(1):e1012812. doi: 10.1371/journal.ppat.1012812 (PMC11750108; doi:10.1371/journal.ppat.1012812)
Supplement: S1 Table — (DOCX) [file ppat.1012812.s012.docx]

**S1 Table: Cryo-ET data from wild type and mutants.**

| **Strain** | **No. of tomograms** | **No. of motors** | **No. of collar subunits** | **Estimated resolution** |
| --- | --- | --- | --- | --- |
| WT | 950 | 5,711 | 91,376 | 1.34nm |
| Δ*bb0236/*Δ*flcD* | 207 | 858 | 13,728 | 4.37nm |
| Δ*flcA* | 179 | 1,286 | 20,576 | 2.32nm |
| Δ*flcB* | 268 | 1,693 | 27,088 | 2.42nm |
| Δ*flcC* | 243 | 2,435 | 38,960 | 2.68nm |
