# Supplementary material for: FlbB forms a distinctive ring essential for periplasmic flagellar assembly and motility in Borrelia burgdorferi
Source: PLoS Pathog. 2025 Jan 8;21(1):e1012812. doi: 10.1371/journal.ppat.1012812 (PMC11750108; doi:10.1371/journal.ppat.1012812)
Supplement: S2 Table — (DOCX) [file ppat.1012812.s013.docx]

**S2 Table: Fitting AlphaFold2-predicted models into cryo-ET maps.**

| **Protein** | **Cross-correlation of model fitting** |
| --- | --- |
| FlbB | 0.7072 |
| FlcB | 0.5840 |
| FlcC | 0.6930 |
| Bb0236/FlcD | 0.6320 |
| FlcA | 0.5586 |
