# Supplementary material for: FlbB forms a distinctive ring essential for periplasmic flagellar assembly and motility in Borrelia burgdorferi
Source: PLoS Pathog. 2025 Jan 8;21(1):e1012812. doi: 10.1371/journal.ppat.1012812 (PMC11750108; doi:10.1371/journal.ppat.1012812)
Supplement: S3 Table — (DOCX) [file ppat.1012812.s014.docx]

**S3 Table: Interactions among collar and stator proteins confirmed experimentally.**

| **Protein 1** | **Protein 2** | **Interaction?^a^** | **Agree?^b^** | **Source** |
| --- | --- | --- | --- | --- |
| FlbB | FlcA | No | Yes | [1] |
|  | FliL | Yes | Yes | [2] |
|  | FlcC | Yes | No | [3] |
|  | Bb0236/FlcD | Yes | Yes | [4] |
|  | FlcB | Yes | Yes | This study |
|  | FlbB | Yes | Yes | [2] |
|  | FliF | Yes | Yes | This study |
| Bb0236/FlcD | FlcC | Yes | Yes | [3] |
|  | FlcB | No | Yes | [3] |
|  | FlcA | No | Yes | [1] |
| FlcB | FlcC | Yes | Yes | [3] |
|  | MotB | No | Yes | [3] |
|  | FliL | No | Yes | [3] |
|  | FlcA | No | Yes | [3] |
| FlcC | MotB | No | Yes | [3] |
|  | FlcA | No | Yes | [3] |
|  | FliL | No | Yes | [3] |
| FliL | MotB | Yes | Yes | [5] |
|  | FlcA | Yes | Yes | [1] |
| FlcA | MotB | Yes | Yes | [1] |

^a^ Whether or not protein 1 and protein 2 interact.

^b^ Whether or not experimental data agree with the predictions of the model.

**References:**

1. Xu H, He J, Liu J, Motaleb MA. BB0326 is responsible for the formation of periplasmic flagellar collar and assembly of the stator complex in *Borrelia burgdorferi*. Mol Microbiol. 2020;113(2):418-29. Epub 20191208. doi: 10.1111/mmi.14428. PubMed PMID: 31743518; PubMed Central PMCID: PMCPMC7178549.

2. Moon KH, Zhao X, Manne A, Wang J, Yu Z, Liu J, et al. Spirochetes flagellar collar protein FlbB has astounding effects in orientation of periplasmic flagella, bacterial shape, motility, and assembly of motors in *Borrelia burgdorferi*. Mol Microbiol. 2016;102(2):336-48. Epub 20160809. doi: 10.1111/mmi.13463. PubMed PMID: 27416872; PubMed Central PMCID: PMCPMC5055450.

3. Chang Y, Xu H, Motaleb MA, Liu J. Characterization of the Flagellar Collar Reveals Structural Plasticity Essential for Spirochete Motility. mBio. 2021;12(6):e0249421. doi: 10.1128/mBio.02494-21. PubMed PMID: 34809456; PubMed Central PMCID: PMCPMC8609358.

4. Moon KH, Zhao X, Xu H, Liu J, Motaleb MA. A tetratricopeptide repeat domain protein has profound effects on assembly of periplasmic flagella, morphology and motility of the lyme disease spirochete *Borrelia burgdorferi*. Mol Microbiol. 2018;110(4):634-47. Epub 20181015. doi: 10.1111/mmi.14121. PubMed PMID: 30303576; PubMed Central PMCID: PMCPMC6218285.

5. Guo S, Xu H, Chang Y, Motaleb MA, Liu J. FliL ring enhances the function of periplasmic flagella. Proc Natl Acad Sci U S A. 2022;119(11):e2117245119. Epub 20220307. doi: 10.1073/pnas.2117245119. PubMed PMID: 35254893; PubMed Central PMCID: PMCPMC8931381.
